# Supplementary material for: UVB Irradiation-Induced Transcriptional Changes in Lignin- and Flavonoid Biosynthesis and Indole/Tryptophan-Auxin-Responsive Genes in Rice Seedlings
Source: Plants (Basel). 2022 Jun 20;11(12):1618. doi: 10.3390/plants11121618 (PMC9229965; doi:10.3390/plants11121618)
Supplement: Supplementary file 1 [file plants-11-01618-s001.zip › plants-1689596-supplementary.pdf]

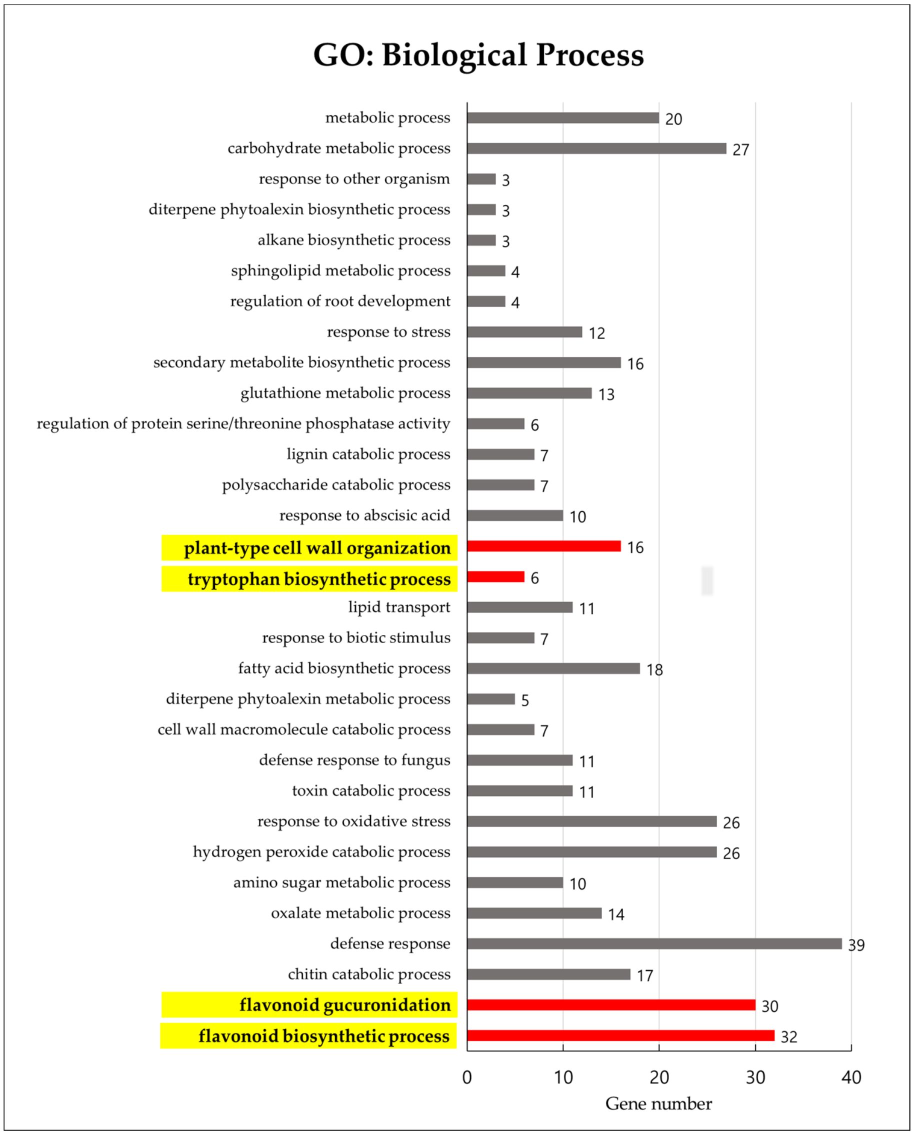

Figure S1. Functional category distribution of selected up-regulated genes assigned by gene ontology (GO) terms to biological processes (DAVID, v 6.8) in +UV-B compared to −UV-B during sink-to-source transition. Red bars (yellow-highlighted) represent the selected genes (84) for further analysis involved in secondary metabolism, cell wall organization (lignin), flavonoid, and indole/tryptophan biosynthesis.

Compound	RT[a]	RRT[b]	Quantification ion[c]
Pyruvic acid	4:45	0.435	174
Lactic acid	4:51	0.444	147
Alanine (Ala)	5:21	0.49	116
Glycolic acid	6:28	0.592	147
Valine (Val)	6:35	0.603	144
Urea	6:50	0.626	189
Ethanolamine	7:06	0.651	174
Phosphoric acid	7:07	0.652	299
Leucine (Leu)	7:08	0.654	158
Isoleucine (Ile)	7:21	0.674	158
Proline (Pro)	7:26	0.681	142
Glycine (Gly)	7:30	0.687	174
Succinic acid	7:34	0.693	147
Glyceric acid	7:40	0.703	147
Fumaric acid	7:55	0.724	245
Serine (Ser)	7:58	0.729	204
Threonine (Thr)	8:12	0.75	219
β-Alanine	8:37	0.789	174
Malic acid	9:06	0.833	147
Aspartic acid	9:22	0.858	100
Methionine (Met)	9:25	0.863	176
Pyroglutamic acid	9:28	0.867	156
4-Aminobutyric acid	9:30	0.87	174
Threonic acid	9:39	0.884	147
Glutamic acid	10:11	0.932	246
Phenylalanine (Phe)	10:19	0.944	218
Xylose	10:24	0.951	103
Asparagine (Asn)	10:35	0.969	116
Glutamine (Gln)	11:22	1.04	156
Shikimic acid	11:33	1.057	204
Citric acid	11:38	1.065	273
Quinic acid	11:54	1.089	345
Fructose	11:58	1.096	103
Mannose	12:04	1.104	147
Galactose	12:07	1.109	147
Glucose	12:09	1.113	160, 319
Inositol	13:27	1.231	305
Ferulic acid	13:33	1.24	338
Tryptophane (Trp)	14:17	1.307	202
Sucrose	16:25	1.502	217

[a]Retention time (min)

[b]Relative retention time (retention time of analyte/retention time of internal standard)

[c]Specific mass ion used for quantification

Figure S2. Extraction and analysis of polar metabolites. Polar metabolites were extracted as described previously (Kim et al., 2016). The metabolites were extracted from powdered tissue (100 mg) by adding 1 mL of 2.5:1:1 (v/v/v) methanol: water: chloroform. Ribitol (60 μL, 0.2 mg/mL) was used an as internal standard (IS). Extraction was performed at 37 °C at a mixing frequency of 1200 rpm for 30 min using a Thermomixer Compact (Eppendorf AG, Germany). The extracts were centrifuged at 16,000 × *g* for 3 min. The polar phase (0.8 mL) mixed with 0.4 mL water was centrifuged at 16,000 × *g* for 3 min. The methanol/water phase was dried in a centrifugal concentrator (CC-105, TOMY, Tokyo, Japan) for 2 h, followed by a freeze dryer for 16 h. MO-derivatization was performed by adding 80 μL of methoxyamine hydrochloride (20 mg/mL) in pyridine and shaking at 30 °C for 90 min. TMS-esterification was performed by adding 80 μL of MSTFA, followed by incubation at 37 °C for 30 min. GC-TOFMS was performed using an Agilent 7890A gas chromatograph (Agilent, Atlanta, GA, USA) coupled to a Pegasus HT-TOF mass spectrometer (LECO, St. Joseph, MI). Each derivatized sample (1 μL) was separated on a 30-cm × 0.25-mm I.D. fused-silica capillary column coated with 0.25-μm CP-SIL 8 CB low bleed (Varian Inc., Palo Alto, CA, USA). The split ratio was set to 1:25. The injector temperature was 230 °C, and a flow rate of helium gas through the column was fixed with 1.0 mL/min. The temperature was set up as follows: initial of 80 °C for 2 min, followed by an increase to 320 °C at 15 °C/min and a 10 min hold at 320 °C. The transfer line temperature and ion-source temperature were 250 and 200 °C, respectively. The scanned mass range was 85–600 *m*/*z*, and the detector voltage was set to 1700 V. ChromaTOF software was used to support peak findings prior to quantitative analysis and for automated deconvolution of the reference mass spectra. NIST and in-house libraries for standard chemicals were utilized for compound identification. The calculations used to quantify the concentrations of all analytes were based on the peak area ratios for each compound relative to the peak area of the IS.

Table S1. Short description of selected up-regulated DEGs (KEGG-indentified) under +UVB.

Pathway	Gene ID	Enzyme	Short Description
Lignin (phenylpropanoid) biosynthesis	Os06g0681600	POD	Heme peroxidase family protein; Similar to peroxidase 39
	Os02g0697400	4CL	4-coumarate:coenzyme A ligase
	Os02g0187800	CAD	Cinnamyl-alcohol dehydrogenase
Flavonoid biosynthesis	Os11g0530600	CHS	Chalcone synthase
	Os03g0819600	CHI	Chalcone isomerase
	Os02g067300	FLS	Flavonol synthase
Phenylalanine, tyrosine and tryptophan Biosynthesis	Os03g0264400	AS	Anthranilate synthase alpha 2 subunit
	Os03g0126000	APRT	Similar to Phosphorybosyl anthranilate transferase 1
	Os02g0266000	PRAI	Similar to N-(5'-phosphoribosyl)anthranilate isomerase
	Os09g0255400	IGPS	Similar to Indole-3-glycerol phosphate synthase, chloroplast precursor
	Os07g0182100	TS	Similar to Tryptophan synthase alpha chain
Auxin-responsive SAUR gene family	*Os06g0701900*	SAUR27	Auxin-responsive SAUR gene family member, SMALL AUXIN-UP RNA 27
	Os09g0547100	SAUR55	Similar to Auxin induced protein, SMALL AUXIN-UP RNA 55
B-box-containing protein	*Os02g0606200*	BBX4	Zinc finger, B-box domain containing protein, B-box-containing protein 4
	Os04g0493000	BBX11	B-box-containing protein 11

Table S2. Primer sequences used for qRT-PCR.

Gene ID	Gene	Forward (5′-3′)	Reverse (5′-3′)	Tm (°C)	Product size (bp)
Os02g0697400	4CL	CATGGTGCTGCTCCAGAA	TAGACGGACTGGGTGAGGAT	57	162
Os02g0187800	CAD	AAGGTGGCCAAGTCGATG	GCACGGTGTCGATGATGTA	57	166
Os06g0681600	POD	GGTGTCGATCAAGCAGGAG	CCTTTCCCGGTGAAGTTGTA	55	189
Os11g0530600	CHS	GGGCTCATCTCGAAGAACAT	CTCGACATGTTGCCGTACTC	55	200
Os03g0819600	CHI	AAGTTCACGAGGGTGACGAT	AGTGGGTGAAGAGGATGGAC	57	193
Os02g0767300	FLS	GCTCTTCCAGGTGGTGAAC	GAGGTCCTTCTGCAGCTTG	57	166
Os03g0264400	AS	AGAGGTTTGAGAGGCGAACA	CCAGCAAGTGGACGGTTAAT	55	178
Os03g0126000	APRT	TGCACGCTGGAAGATCTAAA	GCGTTGTGTTTCCTGTGCTA	55	186
Os02g0266000	PRAI	CGCCATCAATCTCGTCAGTA	AGCAGCTCCATCATTTGGTT	55	161
Os09g025540	IGPS	CGGCATCAATAACCGAAGTT	TTGCAGAAACACCAGCATTC	55	173
Os07g0182100	TS	AGACCGCATTCATTCCATTC	CCTCAAATGTGGTGCCTTTT	55	192
Os06g0701900	SAUR27	GGCGAGCAACAAGATCAG	ATCTCCTCGCCGACGTA	58	135
Os09g0547100	SAUR55	CGAAGGATGGCAGCA	ACGCCAATGGCACCT	60	154
Os02g0606200	BBX4	CAGTTCTCCGACTACGAGAC	GTAGTACGCCACGTCGTT	55	198
Os04g0493000	BBX11	GGTTCAGCTCCGTCTGTAG	ACTCGTAGTCGGAGAGCTG	58	207
Actin		TGTATGCCAGTGGTCGTACC	CCAGCAAGGTCGAGACGAA	57	186

Table S3. Gene expression of −/+UVB treatment by date by RNA-seq.

**Gene**	Gene ID	FPKM						Log2 FC					
		Day 1 −UVB	Day 1 +UVB	Day 3 −UVB	Day 3 +UVB	Day 5 −UVB	Day 5 +UVB	Day 1	*p*-value	Day 3	*p*-value	Day 5	*p*-value
Os02g0697400	4CL	1.96	31.67	31.07	21	25.48	22.63	3.88	0.0063	−0.68	0.4773	−0.32	0.5924
Os02g0187800	CAD	15.45	113.24	130.6	108.04	134.47	179.85	2.74	0.0124	−0.39	0.6093	0.27	0.5671
Os06g0681600	POD	38.62	337.51	214.84	197.21	266.99	360.46	3.00	0.0042	−0.24	0.749	0.29	0.5557
Os11g0530600	CHS	30.74	196.12	243.26	136.85	225.66	244.52	2.54	0.0262	−0.94	0.3774	0.12	0.9593
Os03g0819600	CHI	7.64	823.47	936.42	367.4	900.78	1002.83	6.62	0.001	−1.46	0.094	0.15	0.9869
Os02g0767300	FLS	0	4.53	6.68	2.02	8.02	8.13	2.18	0.0002	−1.84	0.1154	0.02	0.8685
Os03g0264400	AS	74.26	40.74	26.67	256.23	38.49	76.78	−1.00	0.3268	3.15	0.0006	0.85	0.0998
Os03g0126000	APRT	30.72	38.47	20.73	217.45	36.47	75.5	0.19	0.8504	3.28	0.001	0.91	0.0781
Os02g0266000	PRAI	6.01	11.33	4.04	65.1	6.68	21.97	0.79	0.3855	3.9	0.0003	1.57	0.0051
Os09g025540	IGPS	13.36	25.07	1.76	306.51	3.01	71.54	0.78	0.4003	7.33	0.0006	4.43	0.0001
Os07g0182100	TS	29.59	57.8	19.09	490.96	23.22	123.51	0.84	0.3392	4.57	0.0001	2.27	0.0001
Os06g0701900	SAUR27	0.2	0.9	0.88	0	1.39	3.17	2.02	0.4407	−3.14	0.028	1.04	0.3784
Os09g0547100	SAUR55	0	2.65	1.08	0	3.64	2.07	1.41	0.0226	−3.43	0.0155	−0.96	0.4222
Os02g0606200	BBX4	38.75	226.77	337.27	156.8	220.9	92.22	2.42	0.033	−1.22	0.1229	−1.41	0.0064
Os04g0493000	BBX11	1008.35	814.76	1102.5	472.34	793.9	294.49	−0.44	0.6411	−1.34	0.1137	−1.58	0.0034
